# Autocrine Motility Factor-Derived Tetradecapeptides as Dual-Action Chemosensitizers: Bridging Cell Competition Mechanisms with Clinical MDR Reversal in Hematological Malignancies

**DOI:** 10.32604/or.2026.078448

**Published:** 2026-06-16

**Authors:** Changyul Kim, Yu Ju Jung, Da Hee Son, Na Young Kim, Se Gie Kim, Hee Sung Park

**Affiliations:** 1 Department of Health Management, Daegu Catholic University, Gyeongsan, Republic of Korea; 2 Department of Toxicology, Daegu Catholic University, Gyeongsan, Republic of Korea; 3 Department of Cosmetic Science, Kyungsung University, Busan, Republic of Korea

**Keywords:** Autocrine motility factor peptide, drug retention, hematological malignancy, multidrug resistance protein 1 (MDR1), multidrug resistance-associated protein 1 (MRP1)

## Abstract

**Backgrounds:** Autocrine motility factor (AMF) represents a paradoxical protein with dual roles in cancer progression and therapy. This study investigated AMF-derived tetradecapeptides as novel chemosensitizing agents to overcome multidrug resistance (MDR) in hematological malignancies (HMs). **Methods:** Seven AMF variants were screened for anticancer activity across 14 human cancer cell lines using MTT and Cell Counting Kit-8 (CCK-8) assays. Four tetradecapeptides (AMF-derived peptides AAP, HGP, HTP, and SKP) corresponding to the AMF206-219 region were designed and evaluated in three HM cell lines (HL-60, CCRF-CEM, IM-9) for growth inhibition, cellular internalization, reactive oxygen species (ROS) production, mitochondrial membrane potential, and gene/protein expression. Synergism with doxorubicin (DOX) and other chemotherapeutic agents was quantified by combination index (CI) analysis using the Chou-Talalay method and spheroid culture assays. **Results:** AMF variants demonstrated broad-spectrum anticancer activity, with HL-60 acute promyelocytic leukemia cells showing exceptional sensitivity. AMF variants functioned as cell competition-mediated killing signals, converting cancer cells into metabolically compromised “loser” cells through glucose metabolism disruption and oxidative stress induction. Among the four tetradecapeptides, AAP exhibited the most consistent growth inhibition across HM cell lines (HL-60, CCRF-CEM, IM-9) while maintaining gp78/AMFR-mediated cellular internalization. AAP treatment induced dose-dependent ROS production, mitochondrial membrane potential alterations, and p53 upregulation, though glucose-6-phosphate dehydrogenase (G6PD) suppression showed cell-type specificity. Remarkably, AAP demonstrated potent synergistic effects with DOX, reducing IC50 values by 28–53% across tested HM cells through multiple complementary mechanisms: enhanced intracellular DOX retention, elevated oxidative stress, and downregulation of multidrug resistance protein 1 (MDR1; P-glycoprotein) and multidrug resistance-associated protein 1 (MRP1) in specific cell types. AAP similarly synergized with daunorubicin but not with etoposide or cytarabine, suggesting selectivity for ROS-inducing anthracyclines. **Conclusions:** These findings establish AMF-derived peptides as promising human-origin chemosensitizers capable of overcoming multidrug resistance in HMs through multi-targeted mechanisms. The ability to reduce anthracycline dosing while maintaining efficacy offers potential for minimizing cardiotoxicity and other dose-limiting adverse effects, warranting further preclinical development toward clinical translation.

## 1 Introduction

Therapeutic peptides have emerged as a promising class of anticancer agents, encompassing diverse functional categories: cell-penetrating peptides that facilitate intracellular cargo delivery, antimicrobial peptides that induce selective membrane disruption, tumor-homing peptides that recognize overexpressed cancer receptors, pro-apoptotic peptides that trigger programmed cell death, and peptide-drug conjugates that integrate targeted delivery with cytotoxic payloads [1,2]. Despite their therapeutic potential, clinical translation faces substantial barriers including requirements for sophisticated delivery systems, insufficient cancer selectivity, immunogenicity of non-human sequences, and most critically, the emergence of peptide-specific resistance mechanisms [3,4,5,6,7].

HMs—a heterogeneous group of disorders including leukemias, lymphomas, and plasma cell dyscrasias—present formidable therapeutic challenges, particularly due to acquired chemoresistance [8,9]. DOX remains a cornerstone chemotherapeutic agent for these malignancies, offering both clinical efficacy and cost-effectiveness through dual cytotoxic mechanisms: topoisomerase IIα (TOP2A) inhibition and ROS generation [10,11]. However, DOX resistance frequently develops through upregulation of ATP-binding cassette (ABC) efflux transporters, particularly MDR1 and MRP1, along with enhanced antioxidant defense systems that collectively diminish intracellular drug accumulation and cytotoxic efficacy [12,13,14,15,16]. These resistance mechanisms represent major obstacles to successful treatment outcomes in HMs, necessitating novel therapeutic strategies that can simultaneously target cancer cells and overcome multidrug resistance.

AMF represents the secreted isoform of glucose-6-phosphate isomerase (GPI), a glycolytic enzyme with dual functionality [17,18]. Beyond its canonical role in glucose metabolism, extracellular AMF functions as a cytokine that promotes cancer cell motility, proliferation, and metastatic dissemination through activation of downstream signaling cascades via its receptor gp78/AMFR [19,20,21,22]. Paradoxically, AMF also demonstrates selective growth inhibition across diverse cancer cell lines, revealing a dual nature in cancer biology. Guided by principles of cell competition involving paracrine signaling modulation, we previously identified AMF secreted by HeLa cells that specifically inhibited A549 lung adenocarcinoma proliferation [23]. Subsequent investigations revealed that AMF variants cloned from different cancer cell lineages exhibit comparable tumor-selective growth inhibition through mechanisms involving G6PD suppression, oxidative stress induction, and apoptosis activation [24,25,26,27,28]. Most notably, we demonstrated that short AMF-derived peptides retained the antiproliferative activity of full-length AMF proteins, showing efficacy against triple-negative breast cancer cell lines while simultaneously enhancing DOX retention and suppressing MDR1 expression [28].

Building upon these findings, the current study aimed to systematically evaluate AMF peptide activity—both as monotherapy and in combination with chemotherapeutic agents—against a panel of HM cell lines including leukemia and lymphoma models. Specifically, we hypothesized that tetradecapeptides derived from the AMF206-219 region would retain the antiproliferative and chemosensitizing properties of full-length AMF while offering advantages in terms of reduced immunogenicity, enhanced tissue penetration, and improved pharmacokinetic profiles. To test this hypothesis, we designed four tetradecapeptides and characterized their mechanisms of action, focusing on growth inhibition, cellular internalization, metabolic disruption, and chemosensitization properties, with the goal of establishing AMF-derived peptides as promising fourth-generation MDR modulators for hematological malignancies.

## 2 Materials and Methods

### 2.1 Cell Lines and Culture Conditions

Human cancer cell lines including 253J, AsPC-1, DU145, HeLa, Hep 2, Hep G2, A549, HT-29, MDA-MB-231, SKOV3, SNU-484, U-87 MG, and YD-10B, as well as HM cell lines HL-60, CCRF-CEM, and IM-9, were obtained from the Korean Type Culture Collection (KTCC; Seoul, Republic of Korea). All cell lines were authenticated using short tandem repeat profiling within the last six months by the supplier to ensure experimental reliability. Mycoplasma contamination was regularly tested using a PCR-based assay and confirmed negative prior to use. Adherent cell lines were cultured in Dulbecco’s Modified Eagle Medium (DMEM; Cat#SH30022.FS, HyClone™, Logan, UT, USA), while suspension HM cell lines were cultured in RPMI-1640 medium (Cat#SH30011.03, HyClone™). Both media were supplemented with 10% fetal bovine serum (FBS; Cat#10099141, Gibco, Thermo Fisher Scientific, Waltham, MA, USA) and 1% penicillin-streptomycin (Cat#15140122, Gibco). All cell cultures were maintained at 37°C in a humidified incubator with 5% CO_2_.

### 2.2 Proteins and Peptides

AMF variants were cloned from various cancer cell lines, including AA-AMF (A549, GenBank: BC004982), AS-AMF (AsPC-1, GenBank: MW664917), DA-AMF (DU145, GenBank: MW664916), HA-AMF (HeLa, GenBank: KY379509), HG-AMF (Hep G2, GenBank: MW664918), HT-AMF (HT-29, GenBank: MW843569), MA-AMF (MCF-7, GenBank: MW664919), and SK-AMF (SKOV3, GenBank: MW664910). Recombinant AMF proteins were produced using *Escherichia coli* BL21 cells. Briefly, *E. coli* cells harboring AMF cDNA in a pCold DNA vector were induced with 0.5 mM IPTG for 24 h at 15°C (Takara Bio, San Jose, CA, USA). Bacterial cells were lysed on ice for 30 min in extraction buffer (50 mM sodium phosphate pH 8.0, 300 mM NaCl, 10 mM imidazole, and 0.5 mM PMSF) containing 1 mg/mL lysozyme. Samples were sonicated (six 15-s bursts with 15-s cooling periods between bursts), centrifuged at 13,000 rpm for 20 min at 4°C, and cleared lysates were applied to His60 Ni resin affinity chromatography columns (Promega, Madison, WI, USA). AMF proteins were quantified using the Bio-Rad Protein Assay Reagent (Cat#5000006, Bio-Rad Laboratories, Hercules, CA, USA). Synthetic AMF peptides and FITC-labeled AMF peptides were obtained from Biostem (Suwon, Gyeonggi-do, Republic of Korea).

### 2.3 Cell Growth Assay

Human cancer cell lines were seeded in 96-well plates at a density of 10,000 cells/well and incubated for 12 h in DMEM supplemented with 10% FBS. AMF variants were then added at a final concentration of 2 μg/mL, and cells were incubated for an additional 48 h. Following treatment, the culture medium was replaced with serum-free DMEM, and 10 μL of MTT solution (5 mg/mL in PBS; Cat#M6494, Thermo Fisher Scientific) was added to each well containing 100 μL of medium. After 3 h incubation, the culture medium was carefully removed, and 100 μL of DMSO:methanol (1:1, v/v) was added to each well to solubilize the formazan crystals. Absorbance was measured at 570 nm using a SpectraMax iD3 microplate reader (Molecular Devices, San Jose, CA, USA). All experimental conditions were performed in triplicate.

### 2.4 HM Cell Growth Assay

HM cells were plated in 96-well plates at 10,000 cells/well in RPMI-1640 medium containing 10% FBS and treated with AMF variants and AMF peptides either alone or in combination with DOX, etoposide, daunorubicin, or cytarabine. After 48 h incubation at 37°C, cell viability was quantified using the Cell Counting Kit-8 (CCK-8) assay (Cat#CK04, Dojindo Laboratories, Kumamoto, Japan). CCK-8 solution (10 μL) was added to each well containing 100 μL of culture medium and incubated for 3 h. Absorbance was measured at 450 nm using the SpectraMax iD3 microplate reader. All conditions were tested in triplicate.

### 2.5 Spheroid Culture Assay

For 3D spheroid culture assays, HM cells were plated in 96-well U-bottom plates at 3000 cells/well and treated with the same agents as described above. Cells were incubated for 5 days to allow spheroid formation. The resulting spheroids were photographed using a microscope, and spheroid diameters were measured from digital images using NIS-Elements imaging software (Nikon Instruments, Tokyo, Japan).

### 2.6 AMF Peptide Internalization

In cellular internalization assays, FITC-labeled AMF peptides were added to cells (1 × 10^6^ cells/well in 24-well plates, in triplicate). After 24 h incubation, cells were rinsed twice with PBS prior to fluorescence imaging using an Olympus FV3000 confocal microscope (Olympus, Tokyo, Japan).

### 2.7 DOX Fluorescence Assay

In DOX retention assays, HM cells were seeded at 1 × 10^6^ cells/well in 24-well plates and exposed to DOX alone or in combination with AMF peptides. After 24 h incubation, cells were rinsed twice with PBS prior to fluorescence imaging using the Olympus FV3000 confocal microscope. Additionally, rinsed cells were transferred to 96-well black plates with clear glass bottoms for quantitative measurement of DOX retention using the SpectraMax iD3 microplate reader, with excitation and emission wavelengths set at 470 nm and 560 nm, respectively.

### 2.8 ROS Assay

AMF peptides were added to cells (1 × 10^6^ cells/well in 24-well plates, performed in triplicate). After 24 h incubation, cells were treated with 10 μM 2′,7′-dichlorofluorescin diacetate (DCFDA; Cat#287810, Merck, Rahway, NJ, USA) for 1 h, washed twice with PBS, and subjected to fluorescence imaging using the Olympus FV3000 confocal microscope. To quantify time-course ROS production, cells were seeded in 96-well plates at a density of 1 × 10^4^ cells/well and treated with AMF peptides for 8 h, followed by the addition of 10 μM DCFDA. DCFDA fluorescence intensity was measured continuously over 24 h using a BioTek Cytation 7 Cell Imaging Multi-Mode Reader (Agilent, Santa Clara, CA, USA) at excitation and emission wavelengths of 485 nm and 528 nm, respectively.

### 2.9 Mitochondrial Membrane Potential (ΔΨm) Analysis

Cells seeded in 24-well plates at a density of 1 × 10^6^ cells/well were treated with AMF peptides at indicated concentrations. After 24 h, cells were treated with 10 μM rhodamine 123 (Cat#R8004, Sigma-Aldrich, St. Louis, MO, USA) for 30 min at 37°C in the dark, washed twice with PBS, and then resuspended in 100 μL PBS before being transferred to 96-well black plates with clear glass bottoms. Fluorescence intensity was measured using the SpectraMax iD3 microplate reader, with excitation and emission wavelengths set at 485 nm and 535 nm, respectively. Loss of mitochondrial membrane potential was indicated by decreased rhodamine 123 fluorescence intensity.

### 2.10 RT-PCR

RNA extraction was performed using the RNeasy Mini Kit (Cat#74106, Qiagen, Venlo, The Netherlands), followed by reverse transcription with the SuperScript III cDNA Synthesis Kit (Cat#18080051, Invitrogen, Waltham, MA, USA). Quantitative PCR (qPCR) was conducted using Bio-Rad SYBR Green Supermix (Cat#1725272, Bio-Rad Laboratories), and relative mRNA expression levels were calculated using the 2^−ΔΔCt^ method, normalized to β-actin mRNA. The primer sequences were as follows: MDR1 (F: 5′-GAGAGATCCTCACCAAGCGG-3′; R: 5′-ATCATTGGCGAGCCTGGTAG-3′); MRP1 (F: 5′-ATCACAGGGTTGATTGTCCG-3′; R: 5′-GCGCATTCCTTCTTCCAGTT-3′); β-actin (F: 5′-CATGTACGTTGCTATCCAGGC-3′; R: 5′-CTCCTTAATGTCACGCACGAT-3′).

### 2.11 Western Blot Analysis

Total protein samples were separated by sodium dodecyl sulfate-polyacrylamide gel electrophoresis (SDS-PAGE) and transferred to polyvinylidene fluoride (PVDF) membranes. After blocking with 5% skim milk for 1 h, membranes were incubated with primary antibodies overnight at 4°C, followed by 1 h incubation with HRP-conjugated secondary antibodies (anti-mouse IgG-HRP, Cat#sc-516102; anti-rabbit IgG-HRP, Cat#sc-2357; Santa Cruz Biotechnology, Dallas, TX, USA) at room temperature. Protein bands were detected using SuperSignal™ West Pico Chemiluminescent Substrate (Thermo Fisher Scientific) and analyzed using ImageJ software (National Institutes of Health, Bethesda, MD, USA). The primary antibodies used were all purchased from Santa Cruz Biotechnology (Dallas, TX, USA): β-actin (Cat#sc-47778), MDR1 (Cat#sc-390883), MRP1 (Cat#sc-18874), p53 (Cat#sc-126), and G6PD (Cat#sc-373887). Band intensities were quantified by densitometric analysis using ImageJ software, and protein expression levels were normalized to β-actin as a loading control. Relative band intensities are presented as bar graphs below each Western blot image.

### 2.12 Statistical Analysis

Data are presented as the mean ± standard deviation (SD) from three independent experiments. Prior to statistical testing, normality of data distribution was assessed using the Shapiro-Wilk test. Statistical significance was determined using Student’s *t*-test for pairwise comparisons between two groups, or one-way analysis of variance (ANOVA) followed by Tukey’s post hoc test for multiple comparisons. Statistical significance was set at *p* < 0.05. All analyses were performed using GraphPad Prism version 8.03 software (GraphPad Software, Boston, MA, USA).

## 3 Results

### 3.1 AMF Variants Exhibit Broad-Spectrum Anticancer Activity through Cell Competition-Mediated Selective Apoptosis

**Figure 1:**
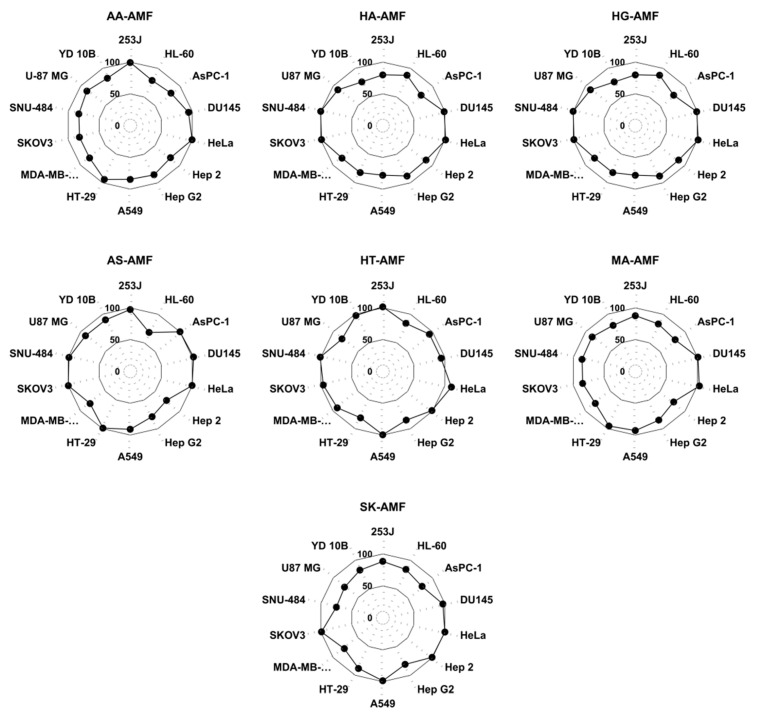
Comparative anticancer activity profiles of AMF variants across diverse cancer cell lines. Radar charts depicting the anticancer efficacy of seven AMF variants (AA-AMF, HA-AMF, HG-AMF, AS-AMF, HT-AMF, MA-AMF, and SK-AMF) across fourteen cancer cell lines. Cell lines tested include 253J (bladder cancer), YD-10B (oral squamous cell carcinoma), HL-60 (acute promyelocytic leukemia), U87 MG (glioblastoma), AsPC-1 (pancreatic adenocarcinoma), SNU-484 (gastric cancer), DU145 (prostate cancer), SKOV3 (ovarian cancer), HeLa (cervical cancer), MDA-MB-231 (triple-negative breast cancer), Hep 2 (laryngeal carcinoma), HT-29 (colorectal adenocarcinoma), Hep G2 (hepatocellular carcinoma), and A549 (non-small cell lung cancer). Cells were treated with 2 μg/mL of each AMF variant for 48 h, and cell viability was assessed by MTT or CCK-8 assay. Radial distance from center (0–100 scale) represents percent cell viability relative to untreated control cells, with values closer to the center indicating stronger growth inhibitory activity. Data represent mean values from three independent experiments. Abbreviations: AA-AMF, autocrine motility factor from A549; HA-AMF, AMF from HeLa; HG-AMF, AMF from Hep G2; AS-AMF, AMF from AsPC-1; HT-AMF, AMF from HT-29; MA-AMF, AMF from MCF-7; SK-AMF, AMF from SKOV3; MTT, 3-(4,5-dimethylthiazol-2-yl)-2,5-diphenyltetrazolium bromide; CCK-8, Cell Counting Kit-8; SD, standard deviation.

To assess the anticancer potential of AMF variants, we evaluated their cytotoxic activity against 14 human cancer cell lines representing diverse tissue origins. Seven AMF variants (AA-AMF, HA-AMF, HG-AMF, AS-AMF, HT-AMF, MA-AMF, and SK-AMF) were tested across this panel, which included HL-60 HM cells and metastatic/solid tumor cells (U-87 MG, AsPC-1, DU145, HeLa, Hep 2, Hep G2, A549, HT-29, MDA-MB-231, SKOV3, SNU-484, YD-10B, 253J). The radar plot profiles revealed that AMF variants functioned as highly aggressive anticancer agents with remarkable pan-cancer activity, with HL-60 acute promyelocytic leukemia cells demonstrating exceptional sensitivity across all seven AMF variants, consistently showing the most pronounced growth inhibition among tested cell lines (Fig. 1). This marked sensitivity positioned HL-60 as an optimal model for investigating AMF-mediated killing mechanisms. Notably, AMF variants demonstrated broad-spectrum anticancer activity by functioning as killing signal factors that target conserved cellular vulnerabilities across diverse malignancies. Consistent with cell competition theory, AMF variants convert cancer cells into metabolically compromised “loser” cells, thereby exploiting glucose metabolism and oxidative stress dependencies to induce selective apoptosis while sparing normal cells [23,26].

### 3.2 AMF Variants Exhibit Cell-Selective Growth Inhibition and Synergistic Chemosensitization in Hematological Malignancy Models

**Figure 2:**
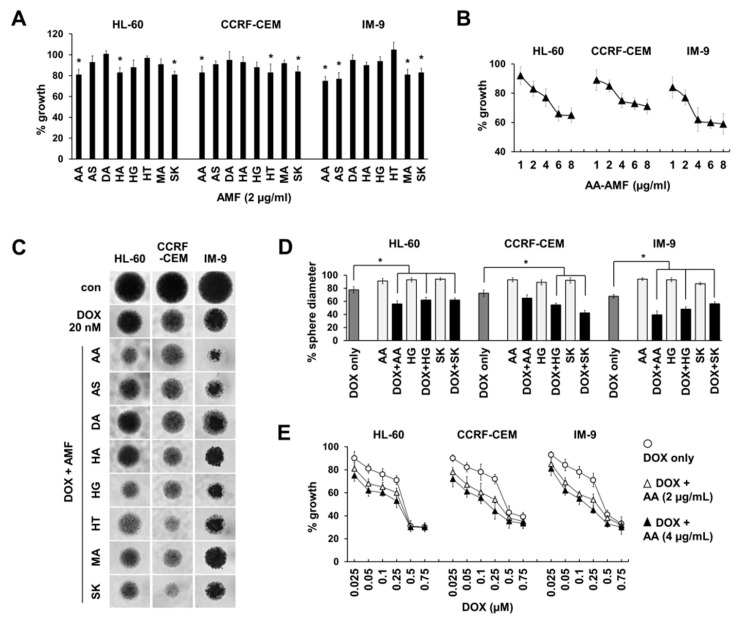
Autocrine motility factor (AMF) variants induce growth inhibition and enhance doxorubicin (DOX) sensitivity in hematological malignancy (HM) cell lines. (**A**) Cell viability of HL-60, CCRF-CEM, and IM-9 cells treated with eight AMF variants (AA, AS, DA, HA, HG, HT, MA, and SK) at 2 μg/mL for 48 h, assessed by CCK-8 assay. Data are presented as percent growth relative to untreated control cells (mean ± SD from three independent experiments; *p < 0.05 vs. control). (**B**) Dose-dependent growth inhibition of HL-60, CCRF-CEM, and IM-9 cells following treatment with AA-AMF at concentrations of 1, 2, 4, 6, and 8 μg/mL for 48 h, assessed by CCK-8 assay. Data represent mean ± SD from three independent experiments. (**C**) Representative images (40× magnification) of spheroid formation showing the combined effects of doxorubicin (DOX, 20 nM) and eight AMF variants on HL-60, CCRF-CEM, and IM-9 cells. Cells were treated with DOX alone or in combination with 2 μg/mL of individual AMF variants, and spheroid formation was assessed after 5 days. (**D**) Quantification of spheroid-forming ability expressed as percent sphere diameter relative to untreated control. Gray bars represent DOX treatment alone (20 nM), and black bars represent DOX combined with each AMF variant. Data show mean ± SD from three independent experiments (*p < 0.05 vs. DOX alone). (**E**) Synergistic growth inhibition of HL-60, CCRF-CEM, and IM-9 cells treated with increasing concentrations of DOX (0.025–0.75 μM) alone (open circles) or in combination with AA-AMF at 2 μg/mL (open triangles) or 4 μg/mL (closed triangles) for 48 h. Cell viability was determined by CCK-8 assay. Data represent mean ± SD from three independent experiments. Abbreviations: DOX, doxorubicin; HM, hematological malignancy; AMF, autocrine motility factor; AA–SK, AMF variant prefixes (cell line of origin); CCK-8, Cell Counting Kit-8; SD, standard deviation.

To further elucidate this selectivity, three HM cell lines were examined: HL-60, CCRF-CEM T-lymphoblastoid cells, and IM-9 B-lymphoblastoid cells. Treatment with eight AMF variants revealed distinct growth inhibition patterns, with AA-AMF and SK-AMF demonstrating the most consistent suppressive effects across all three HM cell lines (Fig. 2A). Dose-response analysis using AA-AMF at concentrations ranging from 1–8 μg/mL showed concentration-dependent growth inhibition in all three HM cell lines, though IC50 values were not achieved within this concentration range (Fig. 2B). These findings underscore the variant-dependent and cell-selective antiproliferative properties of AMF [23,24,25]. The selective activity of AMF variants was further confirmed in three-dimensional spheroid culture studies examining combination treatments with DOX. When 4 μg/mL AMF variants were combined with 20 nM DOX, selective synergistic effects were clearly observed with AA-AMF and SK-AMF across multiple cell lines. This synergistic effect was also evident when HG-AMF was used in HL-60 cells, HT-AMF in CCRF-CEM cells, and AS-AMF or HG-AMF in IM-9 cells (Fig. 2C,D). In 48-h growth assays, 4 μg/mL AA-AMF treatment substantially reduced the IC50 of DOX: from 0.36 to 0.26 μM in HL-60 cells, from 0.52 to 0.28 μM in CCRF-CEM cells, and from 0.48 to 0.29 μM in IM-9 cells (Fig. 2E).

### 3.3 Internalized AMF-Derived Tetradecapeptides Exhibit Type-Dependent Inhibition of Cell Growth

**Figure 3:**
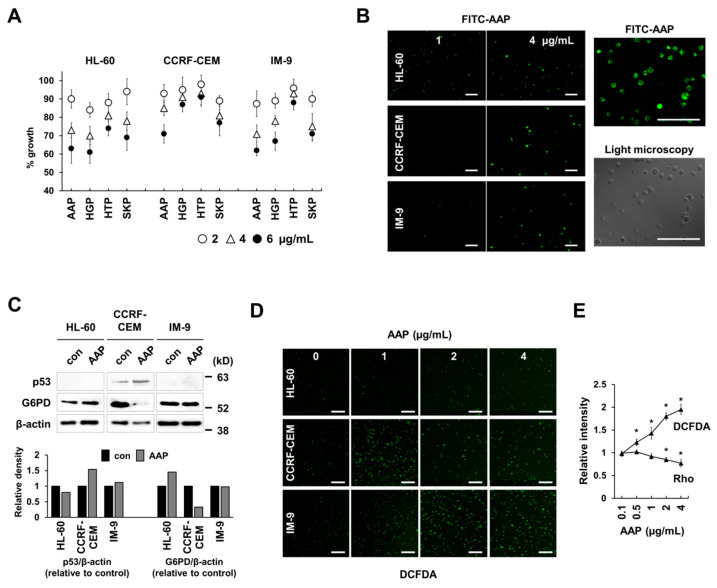
AMF peptides induce growth inhibition in hematological malignancy cells through oxidative stress induction. (**A**) Dose-dependent growth inhibition of HL-60, CCRF-CEM, and IM-9 cells treated with four representative AMF-derived tetradecapeptides (AAP [FIIASKTFTTQETI], HGP [RIIASKTFTTQETI], HTP [FIIASKTFTSQETI], and SKP [FIIASKTFTTQVDI]) at concentrations of 2, 4, and 6 μg/mL for 48 h. Cell viability was assessed by Cell Counting Kit-8 (CCK-8) assay. Data represent mean ± SD from three independent experiments. (**B**) Representative fluorescence microscopy images showing cellular uptake of FITC-conjugated AAP in HL-60, CCRF-CEM, and IM-9 cells treated with 1 or 4 μg/mL for 24 h. Right panels show higher magnification fluorescence and corresponding light microscopy images of FITC-AAP–treated CCRF-CEM cells. Scale bars, 100 μm. (**C**) Western blot analysis of p53 and G6PD protein expression in HL-60, CCRF-CEM, and IM-9 cells treated with AAP (4 μg/mL) for 24 h. β-actin serves as loading control. Quantification of relative band intensities normalized to β-actin is shown below each blot. (**D**) Representative fluorescence microscopy images of intracellular ROS generation in HL-60, CCRF-CEM, and IM-9 cells treated with increasing concentrations of AAP (0, 1, 2, and 4 μg/mL) for 24 h, followed by incubation with 10 μM DCFDA for 30 min. Scale bars, 100 μm. (**E**) Quantification of relative fluorescence intensity for DCFDA and Rhodamine 123 (Rho) in CCRF-CEM cells treated with AAP at 0.1, 0.5, 1, 2, and 4 μg/mL for 24 h. Data represent mean ± SD from three independent experiments (*p < 0.05 vs. untreated control). Abbreviations: AMF, autocrine motility factor; AAP/HGP/HTP/SKP, AMF-derived tetradecapeptides; CCK-8, Cell Counting Kit-8; FITC, fluorescein isothiocyanate; G6PD, glucose-6-phosphate dehydrogenase; DCFDA, 2′,7′-dichlorofluorescin diacetate; SD, standard deviation.

This study investigated specific regions of the AMF protein, particularly the AMF117-288 segment, which is essential for both enzymatic and cytokine functions [29,30,31]. Four AMF206-219 tetradecapeptides were designed based on variant-specific sequences: AAP (FIIASKTFTTQETI, identical among AA-, AS-, DA-, HA-, and MA-AMF variants), HGP (RIIASKTFTTQETI, from HG-AMF), HTP (FIIASKTFTSQETI, from HT-AMF), and SKP (FIIASKTFTTQVDI, from SK-AMF). Growth assays revealed variant-dependent and cell-selective antiproliferative properties, with AAP demonstrating consistent growth inhibition across different HM cell lines at 4 μg/mL compared to other AMF peptides, thereby confirming that AMF’s cytotoxic activity and selectivity are preserved in peptide form (Fig. 3A). Fluorescence microscopy revealed dose-dependent intracellular accumulation of FITC-labeled AAP (Fig. 3B), indicating that the peptide retains key AMF characteristics including gp78/AMFR-mediated internalization and dose-dependent growth inhibition. Western blot analysis showed that AAP downregulated G6PD and upregulated p53 in CCRF-CEM cells, suggesting its role as a versatile apoptosis mediator (Fig. 3C). Notably, HL-60 cells harbor a p53 deletion mutation while IM-9 cells express wild-type p53 [32]. Interestingly, G6PD downregulation occurred exclusively in CCRF-CEM cells, contrasting with previous reports of AMF’s consistent G6PD suppression across various cancer cell lines [27,28] and highlighting challenges in developing unified therapeutic strategies for heterogeneous HMs. Despite this specificity, DCFDA assays demonstrated dose-dependent ROS production across all three cell lines following AAP treatment (Fig. 3D). Furthermore, rhodamine 123 fluorescence was significantly reduced in HL-60 cells upon AAP treatment (Fig. 3E), indicating that altered mitochondrial membrane potential and oxidative stress represent common consequences of AAP exposure across different HM cell types.

### 3.4 AMF Peptides Show Remarkable Synergy with DOX

**Figure 4:**
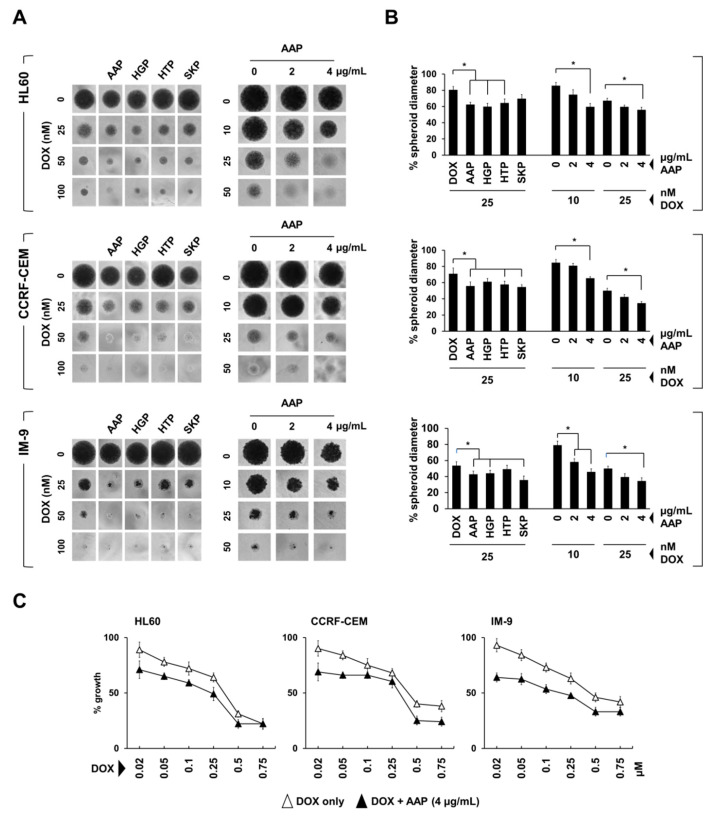
AMF peptides enhance doxorubicin (DOX) sensitivity in hematological malignancy (HM) cells. (**A**) Representative images (40× magnification) of spheroid formation in HL-60, CCRF-CEM, and IM-9 cells treated with AMF peptides (AAP, HGP, HTP, SKP) and DOX at various concentrations for 5 days. Left panels: 4 μg/mL AMF peptides with 0–100 nM DOX. Right panels: 0–4 μg/mL AAP with 0–50 nM DOX. (**B**) Quantification of spheroid diameter. Left: 25 nM DOX combined with individual AMF peptides (4 μg/mL). Right: dose-dependent AAP (0–4 μg/mL) with 10 or 25 nM DOX. Data represent mean ± SD from three independent experiments (*p < 0.05). (**C**) Growth inhibition curves showing synergistic effects of DOX (0.02–0.75 μM) alone (open triangles) versus DOX combined with AAP at 4 μg/mL (closed triangles) in HL-60, CCRF-CEM, and IM-9 cells after 48 h treatment. Cell viability assessed by CCK-8 assay. Data represent mean ± SD from three independent experiments. Abbreviations: AMF, autocrine motility factor; HM, hematological malignancy; DOX, doxorubicin; AAP/HGP/HTP/SKP, AMF-derived tetradecapeptides; CCK-8, Cell Counting Kit-8; SD, standard deviation.

This comprehensive study demonstrates the potent synergistic effects of AMF peptides with DOX across HM cells using spheroid culture assays. The data revealed that all four tested AMF peptides at 4 μg/mL concentration significantly enhanced DOX efficacy at low nanomolar ranges (25–100 nM), as evidenced by spheroid diameter measurements. AAP exhibited dose-dependent synergistic activity, with optimal effects observed at 4 μg/mL when combined with DOX (Fig. 4A,B). IC50 analysis demonstrated substantial chemosensitization across all three cell lines tested (Fig. 4C). Specifically, in 48 h treatment experiments, DOX IC50 values were reduced from 0.43 to 0.31 μM in HL-60 cells (28% reduction), from 0.61 to 0.37 μM in CCRF-CEM cells (39% reduction), and most notably from 0.54 to 0.25 μM in IM-9 cells (53% reduction) when combined with 4 μg/mL AAP. To formally quantify synergism, combination index (CI) values were calculated using the Chou-Talalay method [33] based on the IC50 values of individual agents and their combinations; CI values below 1.0 confirmed synergistic interactions in all three cell lines, corroborating the observed IC50 reductions. The concentration of 4 μg/mL AAP was selected as the primary test dose because dose-response analysis (Fig. 2B and Fig. 3A) showed consistent and maximal antiproliferative activity at this concentration, indicating functional saturation at this dose. These findings suggest promising therapeutic potential for dose-reduction strategies in hematological malignancies, potentially minimizing cardiotoxicity and other DOX-associated adverse effects [34,35].

### 3.5 Synergy Mediated through Enhanced Drug Retention, ROS Production, and Modulation of Drug Resistance Proteins

**Figure 5:**
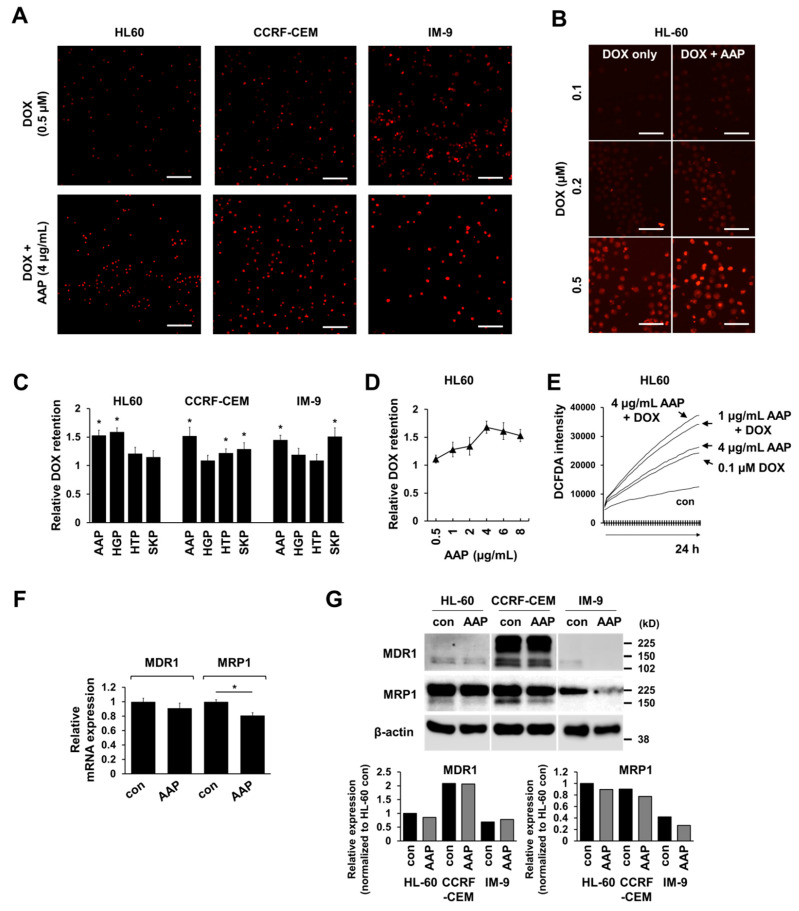
AMF-derived tetradecapeptide AAP enhances intracellular doxorubicin (DOX) retention and suppresses multidrug resistance mechanisms in leukemia cells. (**A**) Representative fluorescence microscopy images showing intracellular DOX accumulation in HL-60, CCRF-CEM, and IM-9 cells after 24 h treatment with DOX (0.5 μM) alone or in combination with AAP (FIIASKTFTTQETI; 4 μg/mL). Scale bars, 100 μm. (**B**) Dose-dependent enhancement of DOX retention in HL-60 cells treated with increasing concentrations of DOX (0.1, 0.2, and 0.5 μM) alone (left panels) or combined with AAP (4 μg/mL, right panels) for 24 h. Scale bars, 50 μm. (**C**) Quantitative analysis of relative DOX retention in HL-60, CCRF-CEM, and IM-9 cells following 24 h treatment with DOX (0.5 μM) combined with AMF-derived peptides (AAP, HGP [RIIASKTFTTQETI], HTP [FIIASKTFTSQETI], or SKP [FIIASKTFTTQVDI] at 4 μg/mL) compared to DOX alone. Data represent mean ± SD from three independent experiments. *p < 0.05 vs. DOX alone. (**D**) Concentration-dependent effect of AAP (0.5–8 μg/mL) on DOX (0.5 μM) retention in HL-60 cells after 24 h treatment, showing saturation of the chemosensitization effect at AAP concentrations ≥4 μg/mL. Data represent mean ± SD from three independent experiments. (**E**) Time-course analysis of reactive oxygen species (ROS) generation measured by 2′,7′-dichlorofluorescin diacetate (DCFDA) fluorescence intensity in HL-60 cells treated with DOX (0.1 μM), AAP (1 or 4 μg/mL), or their combinations over 24 h. (**F**) Relative mRNA expression levels of drug resistance-associated genes multidrug resistance protein 1 (MDR1) and multidrug resistance-associated protein 1 (MRP1) in HL-60 cells following 24 h treatment with AAP (4 μg/mL) compared to control. Data represent mean ± SD from three independent experiments. *p < 0.05 vs. control. (**G**) Western blot analysis of MDR1 and MRP1 protein expression in HL-60, CCRF-CEM, and IM-9 cells after 24 h treatment with AAP (4 μg/mL). Quantification of relative band intensities normalized to β-actin is shown below each blot. β-actin served as loading control. Abbreviations: DOX, doxorubicin; AAP/HGP/HTP/SKP, AMF-derived tetradecapeptides; ROS, reactive oxygen species; DCFDA, 2′,7′-dichlorofluorescin diacetate; MDR1, multidrug resistance protein 1; MRP1, multidrug resistance-associated protein 1; SD, standard deviation.

Fluorescence imaging revealed markedly increased DOX accumulation in HL-60, CCRF-CEM, and IM-9 cells upon co-administration with 4 μg/mL AAP for 24 h (Fig. 5A). This AAP-induced drug retention in HL-60 cells was consistently observed across a DOX concentration range of 0.1–0.5 μM (Fig. 5B). Quantitative analysis of DOX fluorescence intensity demonstrated that AMF peptide variants exhibited differential retention activity across HM cell lines (Fig. 5C), suggesting a correlation between drug retention capacity and chemosensitization efficacy. Notably, AAP’s DOX retention activity in HL-60 cells plateaued at 4 μg/mL, with no further enhancement at higher concentrations (Fig. 5D). Time-dependent DCFDA fluorescence measurements revealed significantly elevated ROS production following combination treatment with DOX and AAP (1 and 4 μg/mL) (Fig. 5E), indicating that oxidative stress contributes substantially to the observed synergistic cytotoxicity. To elucidate the molecular mechanisms underlying enhanced drug retention and chemosensitization, we investigated the effect of AAP on ABC transporters. qRT-PCR analysis demonstrated that AAP treatment significantly downregulated MRP1 in HL-60 cells, while MDR1 expression was moderately decreased (Fig. 5F). Western blot analysis revealed cell-type-specific responses (Fig. 5G). AAP decreased MDR1 expression in HL-60 and IM-9 but not in CCRF-CEM cells. Remarkably, MRP1 protein levels were consistently reduced across all three cell lines. Collectively, these findings demonstrate that AAP exerts synergistic effects with DOX through multiple complementary mechanisms involving enhanced oxidative stress and suppression of drug efflux transporters. The ability of this human-origin peptide to enhance DOX retention by targeting MDR1 and MRP1—key mediators of multidrug resistance and poor clinical prognosis—represents a particularly promising therapeutic strategy.

### 3.6 AMF Peptides Show Synergy with Daunorubicin but Not with Etoposide or Cytarabine

**Figure 6:**
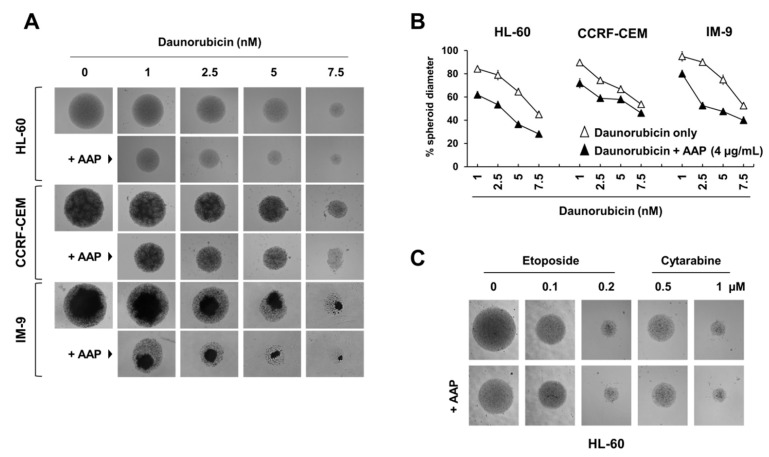
AAP selectively enhances the cytotoxic effects of anthracyclines but not other chemotherapeutic agents in hematological malignancy spheroid models. (**A**) Representative images (40× magnification) of spheroid formation showing dose-dependent effects of daunorubicin (0–7.5 nM) on HL-60, CCRF-CEM, and IM-9 spheroids, either alone (upper rows) or in combination with AAP (4 μg/mL, lower rows). (**B**) Quantitative analysis of spheroid diameter expressed as percentage of control for HL-60, CCRF-CEM, and IM-9 cells treated with increasing concentrations of daunorubicin (1–7.5 nM) alone (open triangles) or combined with AAP (4 μg/mL, filled triangles) for 48 h. Data represent mean ± SD from three independent experiments. (**C**) Representative images (40× magnification) of HL-60 spheroid morphology following treatment with etoposide (0.1 and 0.2 μM) or cytarabine (0.5 and 1 μM), either alone (upper row) or in combination with AAP (4 μg/mL, lower row). Abbreviations: AMF, autocrine motility factor; DOX, doxorubicin; AAP, HGP, HTP, SKP, AMF-derived tetradecapeptides; SD, standard deviation.

To further characterize the chemosensitizing properties of AMF peptides, we investigated their synergistic potential with additional chemotherapeutic agents, including the TOP2A inhibitors daunorubicin and etoposide. Spheroid-based viability assays demonstrated that AAP significantly enhanced daunorubicin-mediated cytotoxicity across all three cell lines (Fig. 6A,B), exhibiting consistent synergistic activity comparable to that observed with DOX. The combination treatment resulted in dramatic, dose-dependent reductions in both spheroid size and viability across a range of daunorubicin concentrations (1–7.5 nM). In contrast, AAP did not exhibit significant synergistic effects when combined with either etoposide (a TOP2A inhibitor) or cytarabine (a nucleoside analog that interferes with DNA synthesis) in HL-60 cells (Fig. 6C) [36]. This differential synergy pattern may reflect distinct mechanisms of action: while DOX, daunorubicin, and cytarabine all induce ROS production, etoposide does not. Since AAP functions primarily through ROS elevation, synergy may require overlapping oxidative stress mechanisms. Additionally, cytarabine’s mechanism involves the incorporation of its active form into DNA to halt replication, potentially explaining limited interaction with AAP’s metabolic targeting approach. These findings suggest that AAP’s chemosensitizing activity is selective for specific ROS-inducing anthracyclines rather than being broadly applicable to all topoisomerase inhibitors.

## 4 Discussion

AMF’s dual nature—functioning as a growth activator in autocrine signaling and a death signal in paracrine competition—provides critical context. This paradoxical behavior reflects structural bifunctionality: dimeric AMF catalyzes glycolysis as GPI, whereas monomeric secreted AMF functions as a cytokine to promote tumor cell motility, metastasis, and cell survival through AMFR/gp78-mediated PI3K/AKT and MAPK/ERK signaling [17,21]. However, the variant-dependent antiproliferative activity suggests AMF-mediated HM cell growth inhibition represents a conserved quality control mechanism exploitable for therapeutic intervention. Cell competition, wherein cells compare fitness and eliminate less fit neighbors through ROS and ATP-dependent pathways, operates as a fundamental surveillance system [37,38]. This study established that exogenous AMF peptides artificially harness this endogenous system, mimicking how winner cells eliminate loser cells through ROS generation, mitochondrial depolarization, and apoptotic activation, thereby representing promising therapeutic agents for HMs through mechanisms combining intrinsic cytotoxicity with chemosensitization. Notably, AMF peptides retaining full-length AMF biological activity offer therapeutic advantages including reduced immunogenicity, enhanced tissue penetration, and improved pharmacokinetics. The human origin particularly minimizes immunogenicity concerns plaguing peptide-based therapies [39,40]. The preservation of gp78/AMFR-mediated cellular internalization in AAP confirms that this 14-amino acid sequence retains essential structural elements for receptor recognition. FITC-labeled AAP demonstrated dose-dependent intracellular accumulation, providing evidence of efficient cellular uptake. Consequently, this receptor-mediated internalization provides tumor-selective homing capability, potentially reducing off-target toxicity to normal hematopoietic cells.

Molecular mechanisms underlying AAP’s anticancer activity reveal both expected and surprising features. AAP-mediated downregulation of G6PD and upregulation of p53 in CCRF-CEM cells confirmed AMF’s metabolic targeting. However, G6PD suppression occurred exclusively in CCRF-CEM cells, highlighting the molecular heterogeneity inherent in HMs. This cell-type specificity contrasts with prior reports of consistent G6PD suppression by full-length AMF proteins across multiple cancer cell lines [27,28], and may reflect differences in basal G6PD expression levels, epigenetic regulation of the G6PD locus, or differential cytokine signaling between lineages. T-lymphoblastoid CCRF-CEM cells may harbor higher intrinsic pentose phosphate pathway activity compared to HL-60 (myeloid) or IM-9 (B-lymphoblastoid) cells, rendering them more susceptible to G6PD-mediated targeting. This metabolic heterogeneity among hematological malignancies underscores challenges in developing unified predictive biomarkers and highlights the importance of characterizing G6PD baseline expression in future patient stratification strategies. Despite this variation, AAP consistently induced dose-dependent ROS production across all cell lines, suggesting that oxidative stress generation operates independently of G6PD modulation. Since G6PD generates NADPH essential for redox homeostasis, its selective suppression explains enhanced ROS accumulation and apoptotic sensitivity [41,42,43]. Meanwhile, reduced rhodamine 123 fluorescence in HL-60 cells confirms that mitochondrial dysfunction contributes to apoptosis across cell types.

The most clinically significant finding is AAP’s remarkable synergy with DOX, reducing IC50 values by 28–53% across tested cell lines. This chemosensitization offers dose-reduction strategies minimizing DOX’s cardiotoxicity, a longstanding challenge in hematological oncology [34,35]. This synergy operates through multiple pathways: enhanced intracellular DOX accumulation (plateauing at 4 μg/mL AAP), elevated ROS levels from combination treatment, and downregulation of MDR1 and MRP1 efflux transporters—well-established mediators of treatment failure [44,45]. The differential effects on efflux transporters merit attention. MRP1 was consistently reduced across all cell types, whereas MDR1 decreased in HL-60 and IM-9 but not CCRF-CEM cells, suggesting that AAP’s chemosensitizing activity may be more universally effective against MRP1-mediated resistance. Although causality between ROS elevation and MDR1/MRP1 downregulation was not directly established in this study, mechanistic evidence suggests a plausible link: ROS can activate NF-κB and AP-1 signaling pathways known to regulate ABC transporter expression [46], and ROS-mediated oxidative damage to transporter proteins may impair their function. Future experiments using ROS scavengers such as N-acetylcysteine (NAC) should be conducted to determine whether abrogation of oxidative stress reverses AAP-induced transporter downregulation and chemosensitization, thereby establishing this causal relationship. Furthermore, AAP’s selectivity provides mechanistic insights and therapeutically valuable properties by demonstrating robust synergy with DOX and daunorubicin, both ROS-inducing anthracyclines, but not with etoposide or cytarabine. Particularly, the selectivity for ROS-inducing agents suggests that synergy requires overlapping oxidative mechanisms. In addition, AAP upregulates p53 in CCRF-CEM cells while maintaining cytotoxicity in p53-null HL-60 cells, confirming engagement of both p53-dependent and p53-independent apoptotic pathways [47,48,49].

From a translational perspective, these findings reveal parallels between developmental quality control and cancer therapeutics. In physiological competition, winner cells induce ROS accumulation in loser cells through pro-inflammatory cytokines, particularly TNF-α, activating JNK and p38 MAPK pathways [50]. AMF peptides artificially impose a “loser” phenotype on cancer cells through similar mechanisms—ROS generation, mitochondrial depolarization, and multi-pathway apoptotic activation—thereby representing a paradigm shift from conventional cytotoxic chemotherapy toward leveraging endogenous tumor suppression mechanisms [51,52]. This approach repurposes evolutionarily conserved surveillance mechanisms for therapeutic cancer elimination.

Clinical translation faces several considerations. The current study has the following limitations, each of which represents an important direction for future work:

(1) All experiments were performed exclusively *in vitro* using established cancer cell lines. Clinical efficacy claims must therefore be interpreted with appropriate caution, and *in vivo* validation in syngeneic or patient-derived xenograft (PDX) models is required before any translational conclusions can be drawn.
(2) The absence of normal hematopoietic control cells (e.g., peripheral blood mononuclear cells [PBMCs] or CD34^+^ progenitors) precludes definitive conclusions about cancer selectivity and clinical safety margins. Future studies must include normal hematopoietic cells to establish a therapeutic window.
(3) Causality between reactive oxygen species (ROS) elevation and MDR1/MRP1 downregulation was not directly established. Future experiments using ROS scavengers such as N-acetylcysteine (NAC) are needed to confirm that abrogating oxidative stress reverses AAP-induced transporter downregulation and chemosensitization.
(4) Tetradecapeptides are subject to proteolytic degradation *in vivo*, and AAP may require structural modifications—such as stapling, cyclization, or PEGylation—to achieve adequate plasma stability and bioavailability. Dedicated pharmacokinetic studies establishing peptide stability, clearance rates, and tissue distribution are essential next steps.
(5) The observed G6PD suppression was restricted to CCRF-CEM cells, limiting the generalizability of the G6PD-NADPH-ROS axis as a universal biomarker for AAP responsiveness across heterogeneous HM subtypes. Future patient stratification strategies should characterize baseline G6PD expression and pentose phosphate pathway activity across HM subtypes.

Despite these limitations, the ability to reduce DOX IC50 values by up to 53% represents a clinically meaningful chemosensitization effect, suggesting that combination therapy could achieve equivalent efficacy at substantially lower anthracycline doses, potentially mitigating cumulative cardiotoxicity. Future investigations should evaluate AMF peptide efficacy in PDX models, assess cardioprotective effects with reduced DOX doses, and examine whether AMF peptides can overcome chemoresistance in relapsed/refractory cases where ABC transporter overexpression drives treatment failure.

## 5 Conclusion

AMF-derived tetradecapeptides represent a novel therapeutic class leveraging cell competition biology to achieve selective cancer cell elimination while reversing multidrug resistance. The dual-action mechanism—intrinsic cytotoxicity combined with chemosensitization through enhanced drug retention and ROS amplification—positions these peptides as promising adjuvants. This biomimetic approach, combined with human origin minimizing immunogenicity, tumor-selective AMFR-mediated homing, and multi-pathway mechanisms, warrants preclinical evaluation in patient-derived xenograft models and early-phase clinical trials for relapsed or refractory hematological malignancies.

## Data Availability

All data or analysis during this study are included in this manuscript.
